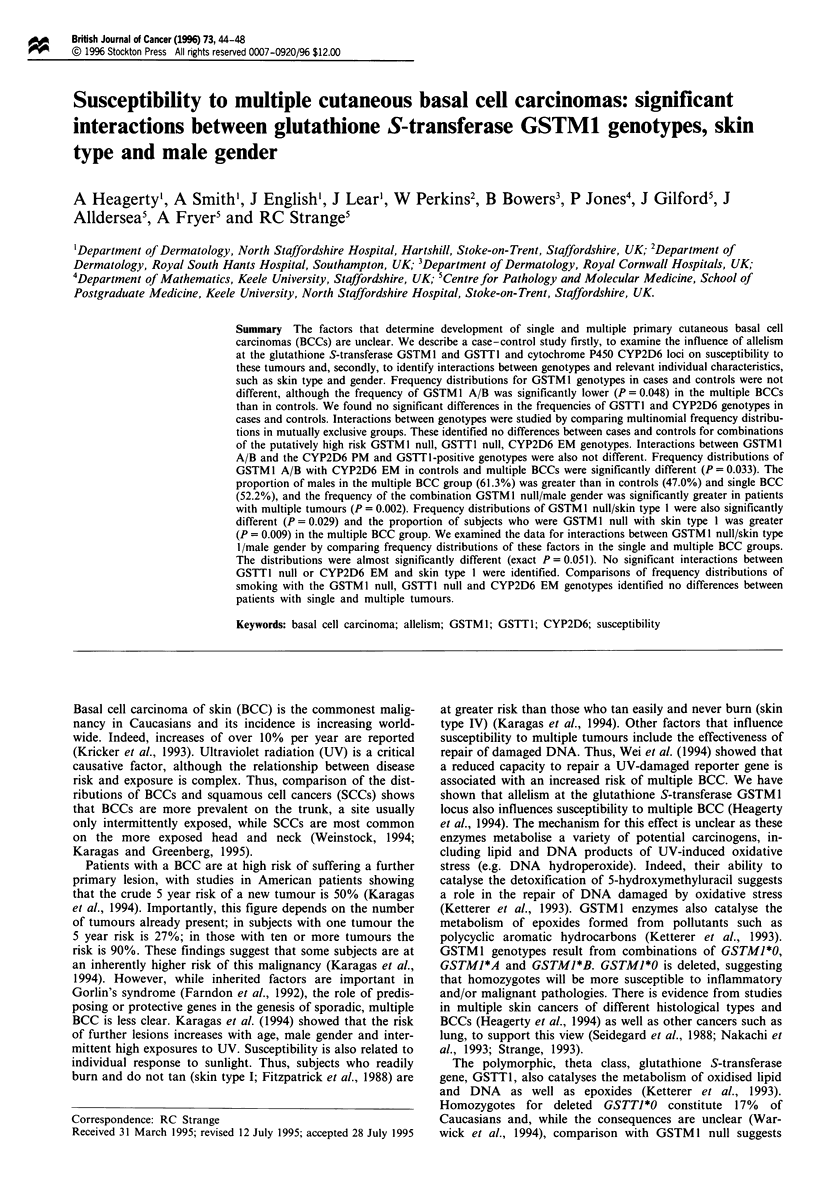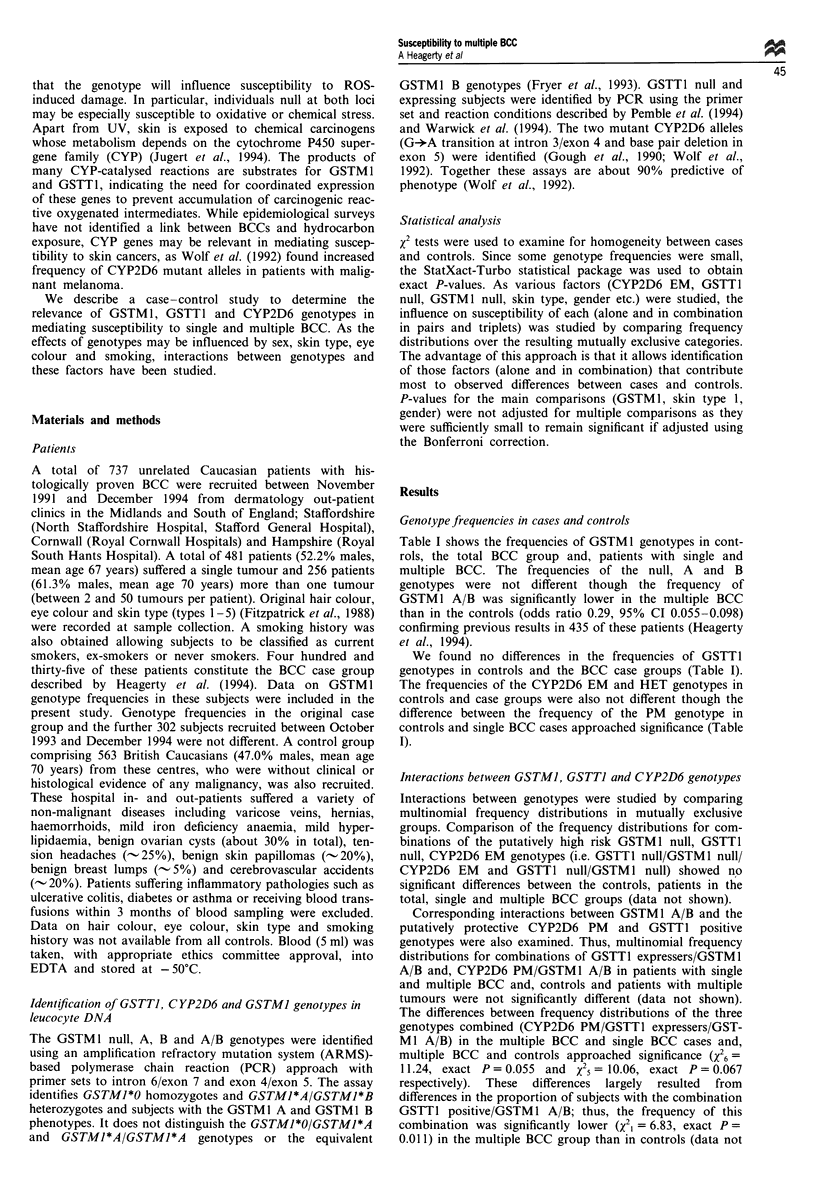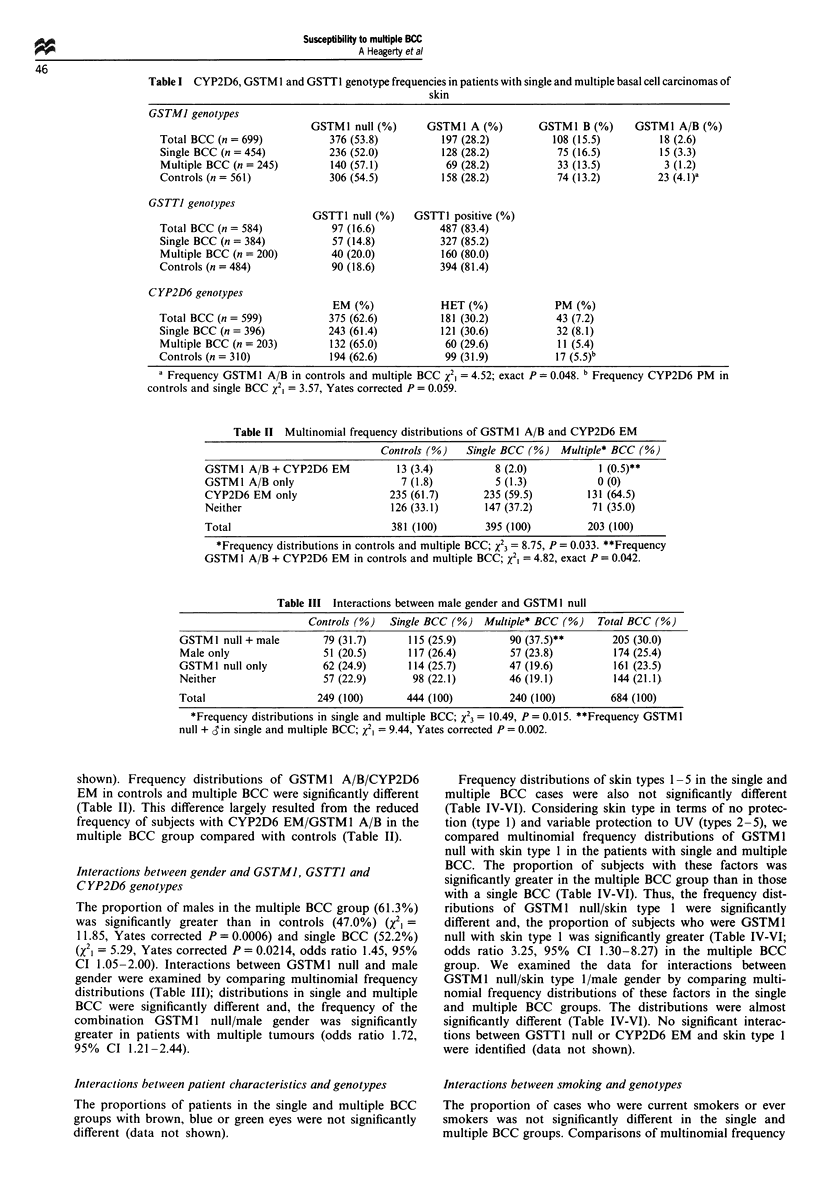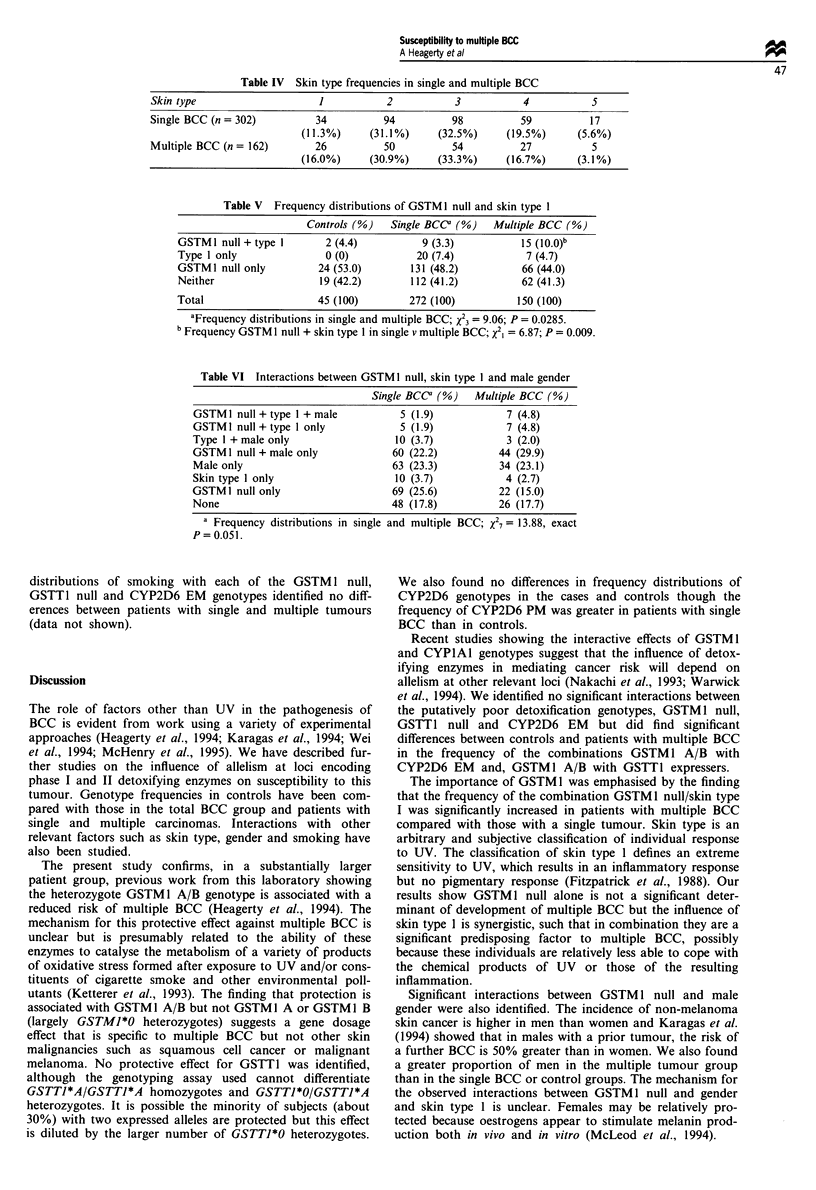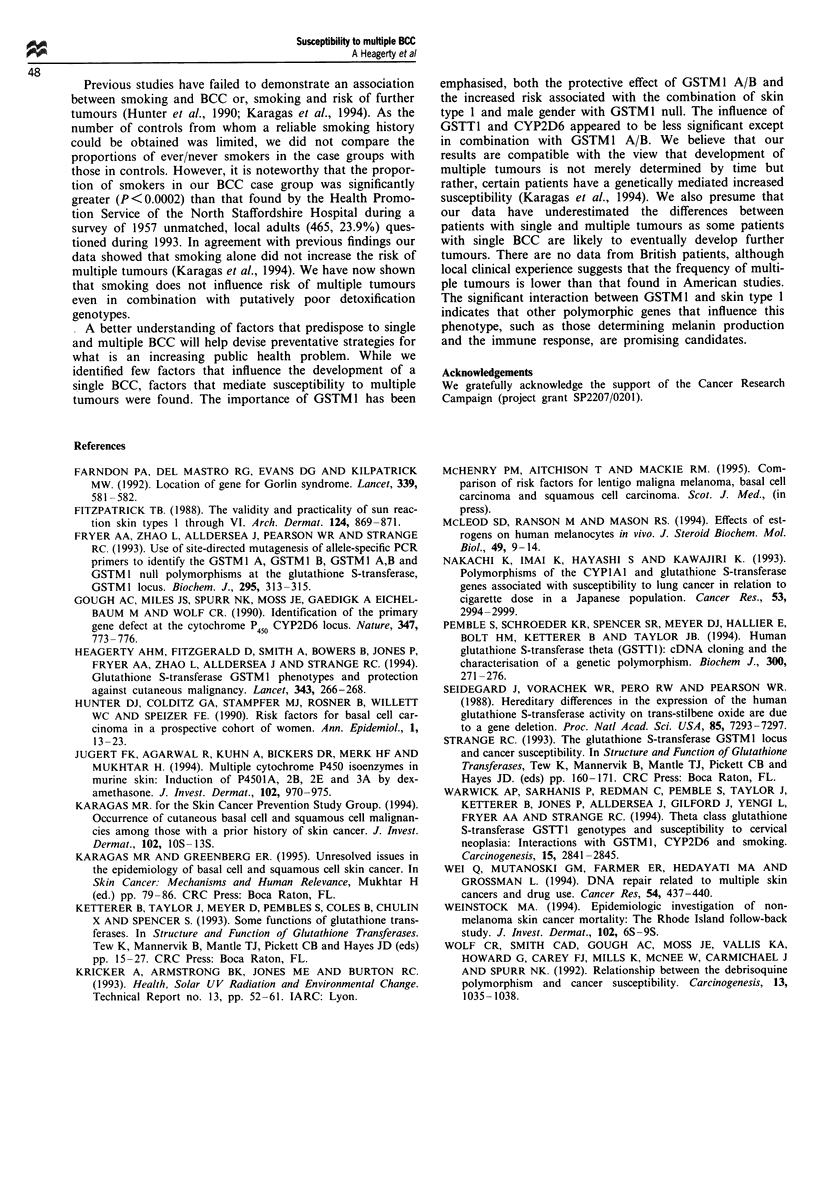# Susceptibility to multiple cutaneous basal cell carcinomas: significant interactions between glutathione S-transferase GSTM1 genotypes, skin type and male gender.

**DOI:** 10.1038/bjc.1996.8

**Published:** 1996-01

**Authors:** A. Heagerty, A. Smith, J. English, J. Lear, W. Perkins, B. Bowers, P. Jones, J. Gilford, J. Alldersea, A. Fryer, R. C. Strange

**Affiliations:** Department of Dermatology, North Staffordshire Hospital, Hartshill, Stoke-on-Trent, UK.

## Abstract

The factors that determine development of single and multiple primary cutaneous basal cell carcinomas (BCCs) are unclear. We describe a case-control study firstly, to examine the influence of allelism at the glutathione S-transferase GSTM1 and GSTT1 and cytochrome P450 CYP2D6 loci on susceptibility to these tumours and, secondly, to identify interactions between genotypes and relevant individual characteristics, such as skin type and gender. Frequency distributions for GSTM1 genotypes in cases and controls were not different, although the frequency of GSTM1 A/B was significantly lower (P = 0.048) in the multiple BCCs than in controls. We found no significant differences in the frequencies of GSTT1 and CYP2D6 genotypes in cases and controls. Interactions between genotypes were studied by comparing multinomial frequency distributions in mutually exclusive groups. These identified no differences between cases and controls for combinations of the putatively high risk GSTM1 null, GSTT1 null, CYP2D6 EM genotypes. Interactions between GSTM1 A/B and the CYP2D6 PM and GSTT1-positive genotypes were also not different. Frequency distributions of GSTM1 A/B with CYP2D6 EM in controls and multiple BCCs were significantly different (P = 0.033). The proportion of males in the multiple BCC group (61.3%) was greater than in controls (47.0%) and single BCC (52.2%), and the frequency of the combination GSTM1 null/male gender was significantly greater in patients with multiple tumours (P = 0.002). Frequency distributions of GSTM1 null/skin type 1 were also significantly different (P = 0.029) and the proportion of subjects who were GSTM1 null with skin type 1 was greater (P = 0.009) in the multiple BCC group. We examined the data for interactions between GSTM1 null/skin type 1/male gender by comparing frequency distributions of these factors in the single and multiple BCC groups. The distributions were almost significantly different (exact P = 0.051). No significant interactions between GSTT1 null or CYP2D6 EM and skin type 1 were identified. Comparisons of frequency distributions of smoking with the GSTM1 null, GSTT1 null and CYP2D6 EM genotypes identified no differences between patients with single and multiple tumours.


British Journal of Cancer (1996) 73, 44-48
© 1996 Stockton Press All rights reserved 0007-0920/96 $12.00

### Susceptibility to multiple cutaneous basal cell carcinomas: significant interactions between glutathione *S*-transferase GSTM1 genotypes, skin type and male gender

A Heagerty[1], A Smith[1], J English[1], J Lear[1], W Perkins[2], B Bowers[3], P Jones[4], J Gilford[5], J Alldersea[5], A Fryer[5] and RC Strange[5]

[1]*Department of Dermatology, North Staffordshire Hospital, Hartshill, Stoke-on-Trent, Staffordshire, UK;* [2]*Department of Dermatology, Royal South Hants Hospital, Southampton, UK;* [3]*Department of Dermatology, Royal Cornwall Hospitals, UK;* [4]*Department of Mathematics, Keele University, Staffordshire, UK;* [5]*Centre for Pathology and Molecular Medicine, School of Postgraduate Medicine, Keele University, North Staffordshire Hospital, Stoke-on-Trent, Staffordshire, UK.*

**Summary** The factors that determine development of single and multiple primary cutaneous basal cell carcinomas (BCCs) are unclear. We describe a case–control study firstly, to examine the influence of allelism at the glutathione *S*-transferase GSTM1 and GSTT1 and cytochrome P450 CYP2D6 loci on susceptibility to these tumours and, secondly, to identify interactions between genotypes and relevant individual characteristics, such as skin type and gender. Frequency distributions for GSTM1 genotypes in cases and controls were not different, although the frequency of GSTM1 A/B was significantly lower ($P = 0.048$) in the multiple BCCs than in controls. We found no significant differences in the frequencies of GSTT1 and CYP2D6 genotypes in cases and controls. Interactions between genotypes were studied by comparing multinomial frequency distributions in mutually exclusive groups. These identified no differences between cases and controls for combinations of the putatively high risk GSTM1 null, GSTT1 null, CYP2D6 EM genotypes. Interactions between GSTM1 A/B and the CYP2D6 PM and GSTT1-positive genotypes were also not different. Frequency distributions of GSTM1 A/B with CYP2D6 EM in controls and multiple BCCs were significantly different ($P = 0.033$). The proportion of males in the multiple BCC group (61.3%) was greater than in controls (47.0%) and single BCC (52.2%), and the frequency of the combination GSTM1 null/male gender was significantly greater in patients with multiple tumours ($P = 0.002$). Frequency distributions of GSTM1 null/skin type 1 were also significantly different ($P = 0.029$) and the proportion of subjects who were GSTM1 null with skin type 1 was greater ($P = 0.009$) in the multiple BCC group. We examined the data for interactions between GSTM1 null/skin type 1/male gender by comparing frequency distributions of these factors in the single and multiple BCC groups. The distributions were almost significantly different (exact $P = 0.051$). No significant interactions between GSTT1 null or CYP2D6 EM and skin type 1 were identified. Comparisons of frequency distributions of smoking with the GSTM1 null, GSTT1 null and CYP2D6 EM genotypes identified no differences between patients with single and multiple tumours.

**Keywords:** basal cell carcinoma; allelism; GSTM1; GSTT1; CYP2D6; susceptibility

Basal cell carcinoma of skin (BCC) is the commonest malignancy in Caucasians and its incidence is increasing worldwide. Indeed, increases of over 10% per year are reported (Kricker *et al.*, 1993). Ultraviolet radiation (UV) is a critical causative factor, although the relationship between disease risk and exposure is complex. Thus, comparison of the distributions of BCCs and squamous cell cancers (SCCs) shows that BCCs are more prevalent on the trunk, a site usually only intermittently exposed, while SCCs are most common on the more exposed head and neck (Weinstock, 1994; Karagas and Greenberg, 1995).

Patients with a BCC are at high risk of suffering a further primary lesion, with studies in American patients showing that the crude 5 year risk of a new tumour is 50% (Karagas *et al.*, 1994). Importantly, this figure depends on the number of tumours already present; in subjects with one tumour the 5 year risk is 27%; in those with ten or more tumours the risk is 90%. These findings suggest that some subjects are at an inherently higher risk of this malignancy (Karagas *et al.*, 1994). However, while inherited factors are important in Gorlin's syndrome (Farndon *et al.*, 1992), the role of predisposing or protective genes in the genesis of sporadic, multiple BCC is less clear. Karagas *et al.* (1994) showed that the risk of further lesions increases with age, male gender and intermittent high exposures to UV. Susceptibility is also related to individual response to sunlight. Thus, subjects who readily burn and do not tan (skin type I; Fitzpatrick *et al.*, 1988) are at greater risk than those who tan easily and never burn (skin type IV) (Karagas *et al.*, 1994). Other factors that influence susceptibility to multiple tumours include the effectiveness of repair of damaged DNA. Thus, Wei *et al.* (1994) showed that a reduced capacity to repair a UV-damaged reporter gene is associated with an increased risk of multiple BCC. We have shown that allelism at the glutathione *S*-transferase GSTM1 locus also influences susceptibility to multiple BCC (Heagerty *et al.*, 1994). The mechanism for this effect is unclear as these enzymes metabolise a variety of potential carcinogens, including lipid and DNA products of UV-induced oxidative stress (e.g. DNA hydroperoxide). Indeed, their ability to catalyse the detoxification of 5-hydroxymethyluracil suggests a role in the repair of DNA damaged by oxidative stress (Ketterer *et al.*, 1993). GSTM1 enzymes also catalyse the metabolism of epoxides formed from pollutants such as polycyclic aromatic hydrocarbons (Ketterer *et al.*, 1993). GSTM1 genotypes result from combinations of *GSTM1*0*, *GSTM1*A* and *GSTM1*B*. *GSTM1*0* is deleted, suggesting that homozygotes will be more susceptible to inflammatory and/or malignant pathologies. There is evidence from studies in multiple skin cancers of different histological types and BCCs (Heagerty *et al.*, 1994) as well as other cancers such as lung, to support this view (Seidegard *et al.*, 1988; Nakachi *et al.*, 1993; Strange, 1993).

The polymorphic, theta class, glutathione *S*-transferase gene, GSTT1, also catalyses the metabolism of oxidised lipid and DNA as well as epoxides (Ketterer *et al.*, 1993). Homozygotes for deleted *GSTT1*0* constitute 17% of Caucasians and, while the consequences are unclear (Warwick *et al.*, 1994), comparison with GSTM1 null suggests

Correspondence: RC Strange
Received 31 March 1995; revised 12 July 1995; accepted 28 July 1995

that the genotype will influence susceptibility to ROS-induced damage. In particular, individuals null at both loci may be especially susceptible to oxidative or chemical stress. Apart from UV, skin is exposed to chemical carcinogens whose metabolism depends on the cytochrome P450 supergene family (CYP) (Jugert et al., 1994). The products of many CYP-catalysed reactions are substrates for GSTM1 and GSTT1, indicating the need for coordinated expression of these genes to prevent accumulation of carcinogenic reactive oxygenated intermediates. While epidemiological surveys have not identified a link between BCCs and hydrocarbon exposure, CYP genes may be relevant in mediating susceptibility to skin cancers, as Wolf et al. (1992) found increased frequency of CYP2D6 mutant alleles in patients with malignant melanoma.

We describe a case–control study to determine the relevance of GSTM1, GSTT1 and CYP2D6 genotypes in mediating susceptibility to single and multiple BCC. As the effects of genotypes may be influenced by sex, skin type, eye colour and smoking, interactions between genotypes and these factors have been studied.

### Materials and methods

### *Patients*

A total of 737 unrelated Caucasian patients with histologically proven BCC were recruited between November 1991 and December 1994 from dermatology out-patient clinics in the Midlands and South of England; Staffordshire (North Staffordshire Hospital, Stafford General Hospital), Cornwall (Royal Cornwall Hospitals) and Hampshire (Royal South Hants Hospital). A total of 481 patients (52.2% males, mean age 67 years) suffered a single tumour and 256 patients (61.3% males, mean age 70 years) more than one tumour (between 2 and 50 tumours per patient). Original hair colour, eye colour and skin type (types 1–5) (Fitzpatrick et al., 1988) were recorded at sample collection. A smoking history was also obtained allowing subjects to be classified as current smokers, ex-smokers or never smokers. Four hundred and thirty-five of these patients constitute the BCC case group described by Heagerty et al. (1994). Data on GSTM1 genotype frequencies in these subjects were included in the present study. Genotype frequencies in the original case group and the further 302 subjects recruited between October 1993 and December 1994 were not different. A control group comprising 563 British Caucasians (47.0% males, mean age 70 years) from these centres, who were without clinical or histological evidence of any malignancy, was also recruited. These hospital in- and out-patients suffered a variety of non-malignant diseases including varicose veins, hernias, haemorrhoids, mild iron deficiency anaemia, mild hyperlipidaemia, benign ovarian cysts (about 30% in total), tension headaches (~25%), benign skin papillomas (~20%), benign breast lumps (~5%) and cerebrovascular accidents (~20%). Patients suffering inflammatory pathologies such as ulcerative colitis, diabetes or asthma or receiving blood transfusions within 3 months of blood sampling were excluded. Data on hair colour, eye colour, skin type and smoking history was not available from all controls. Blood (5 ml) was taken, with appropriate ethics committee approval, into EDTA and stored at −50°C.

### *Identification of GSTT1, CYP2D6 and GSTM1 genotypes in leucocyte DNA*

The GSTM1 null, A, B and A/B genotypes were identified using an amplification refractory mutation system (ARMS)-based polymerase chain reaction (PCR) approach with primer sets to intron 6/exon 7 and exon 4/exon 5. The assay identifies *GSTM1*0* homozygotes and *GSTM1*A/GSTM1*B* heterozygotes and subjects with the GSTM1 A and GSTM1 B phenotypes. It does not distinguish the *GSTM1*0/GSTM1*A* and *GSTM1*A/GSTM1*A* genotypes or the equivalent GSTM1 B genotypes (Fryer et al., 1993). GSTT1 null and expressing subjects were identified by PCR using the primer set and reaction conditions described by Pemble et al. (1994) and Warwick et al. (1994). The two mutant CYP2D6 alleles (G→A transition at intron 3/exon 4 and base pair deletion in exon 5) were identified (Gough et al., 1990; Wolf et al., 1992). Together these assays are about 90% predictive of phenotype (Wolf et al., 1992).

### *Statistical analysis*

$\chi^2$ tests were used to examine for homogeneity between cases and controls. Since some genotype frequencies were small, the StatXact-Turbo statistical package was used to obtain exact *P*-values. As various factors (CYP2D6 EM, GSTT1 null, GSTM1 null, skin type, gender etc.) were studied, the influence on susceptibility of each (alone and in combination in pairs and triplets) was studied by comparing frequency distributions over the resulting mutually exclusive categories. The advantage of this approach is that it allows identification of those factors (alone and in combination) that contribute most to observed differences between cases and controls. *P*-values for the main comparisons (GSTM1, skin type 1, gender) were not adjusted for multiple comparisons as they were sufficiently small to remain significant if adjusted using the Bonferroni correction.

### Results

### *Genotype frequencies in cases and controls*

Table I shows the frequencies of GSTM1 genotypes in controls, the total BCC group and, patients with single and multiple BCC. The frequencies of the null, A and B genotypes were not different though the frequency of GSTM1 A/B was significantly lower in the multiple BCC than in the controls (odds ratio 0.29, 95% CI 0.055–0.098) confirming previous results in 435 of these patients (Heagerty et al., 1994).

We found no differences in the frequencies of GSTT1 genotypes in controls and the BCC case groups (Table I). The frequencies of the CYP2D6 EM and HET genotypes in controls and case groups were also not different though the difference between the frequency of the PM genotype in controls and single BCC cases approached significance (Table I).

### *Interactions between GSTM1, GSTT1 and CYP2D6 genotypes*

Interactions between genotypes were studied by comparing multinomial frequency distributions in mutually exclusive groups. Comparison of the frequency distributions for combinations of the putatively high risk GSTM1 null, GSTT1 null, CYP2D6 EM genotypes (i.e. GSTT1 null/GSTM1 null/CYP2D6 EM and GSTT1 null/GSTM1 null) showed no significant differences between the controls, patients in the total, single and multiple BCC groups (data not shown).

Corresponding interactions between GSTM1 A/B and the putatively protective CYP2D6 PM and GSTT1 positive genotypes were also examined. Thus, multinomial frequency distributions for combinations of GSTT1 expressers/GSTM1 A/B and, CYP2D6 PM/GSTM1 A/B in patients with single and multiple BCC and, controls and patients with multiple tumours were not significantly different (data not shown). The differences between frequency distributions of the three genotypes combined (CYP2D6 PM/GSTT1 expressers/GSTM1 A/B) in the multiple BCC and single BCC cases and, multiple BCC and controls approached significance ($\chi^2_6 = 11.24$, exact $P = 0.055$ and $\chi^2_5 = 10.06$, exact $P = 0.067$ respectively). These differences largely resulted from differences in the proportion of subjects with the combination GSTT1 positive/GSTM1 A/B; thus, the frequency of this combination was significantly lower ($\chi^2_1 = 6.83$, exact $P = 0.011$) in the multiple BCC group than in controls (data not

**Table I** CYP2D6, GSTM1 and GSTT1 genotype frequencies in patients with single and multiple basal cell carcinomas of skin

| | | | | |
|---|---|---|---|---|
| *GSTM1 genotypes* | | | | |
| | GSTM1 null (%) | GSTM1 A (%) | GSTM1 B (%) | GSTM1 A/B (%) |
| Total BCC ($n = 699$) | 376 (53.8) | 197 (28.2) | 108 (15.5) | 18 (2.6) |
| Single BCC ($n = 454$) | 236 (52.0) | 128 (28.2) | 75 (16.5) | 15 (3.3) |
| Multiple BCC ($n = 245$) | 140 (57.1) | 69 (28.2) | 33 (13.5) | 3 (1.2) |
| Controls ($n = 561$) | 306 (54.5) | 158 (28.2) | 74 (13.2) | 23 (4.1)[a] |
| *GSTT1 genotypes* | | | | |
| | GSTT1 null (%) | GSTT1 positive (%) | | |
| Total BCC ($n = 584$) | 97 (16.6) | 487 (83.4) | | |
| Single BCC ($n = 384$) | 57 (14.8) | 327 (85.2) | | |
| Multiple BCC ($n = 200$) | 40 (20.0) | 160 (80.0) | | |
| Controls ($n = 484$) | 90 (18.6) | 394 (81.4) | | |
| *CYP2D6 genotypes* | | | | |
| | EM (%) | HET (%) | PM (%) | |
| Total BCC ($n = 599$) | 375 (62.6) | 181 (30.2) | 43 (7.2) | |
| Single BCC ($n = 396$) | 243 (61.4) | 121 (30.6) | 32 (8.1) | |
| Multiple BCC ($n = 203$) | 132 (65.0) | 60 (29.6) | 11 (5.4) | |
| Controls ($n = 310$) | 194 (62.6) | 99 (31.9) | 17 (5.5)[b] | |

[a] Frequency GSTM1 A/B in controls and multiple BCC $\chi^2_1 = 4.52$; exact $P = 0.048$. [b] Frequency CYP2D6 PM in controls and single BCC $\chi^2_1 = 3.57$, Yates corrected $P = 0.059$.

**Table II** Multinomial frequency distributions of GSTM1 A/B and CYP2D6 EM

| | *Controls (%)* | *Single BCC (%)* | *Multiple* BCC (%)* |
|---|---|---|---|
| GSTM1 A/B + CYP2D6 EM | 13 (3.4) | 8 (2.0) | 1 (0.5)** |
| GSTM1 A/B only | 7 (1.8) | 5 (1.3) | 0 (0) |
| CYP2D6 EM only | 235 (61.7) | 235 (59.5) | 131 (64.5) |
| Neither | 126 (33.1) | 147 (37.2) | 71 (35.0) |
| Total | 381 (100) | 395 (100) | 203 (100) |

*Frequency distributions in controls and multiple BCC; $\chi^2_3 = 8.75$, $P = 0.033$. **Frequency GSTM1 A/B + CYP2D6 EM in controls and multiple BCC; $\chi^2_1 = 4.82$, exact $P = 0.042$.

**Table III** Interactions between male gender and GSTM1 null

| | *Controls (%)* | *Single BCC (%)* | *Multiple* BCC (%)* | *Total BCC (%)* |
|---|---|---|---|---|
| GSTM1 null + male | 79 (31.7) | 115 (25.9) | 90 (37.5)** | 205 (30.0) |
| Male only | 51 (20.5) | 117 (26.4) | 57 (23.8) | 174 (25.4) |
| GSTM1 null only | 62 (24.9) | 114 (25.7) | 47 (19.6) | 161 (23.5) |
| Neither | 57 (22.9) | 98 (22.1) | 46 (19.1) | 144 (21.1) |
| Total | 249 (100) | 444 (100) | 240 (100) | 684 (100) |

*Frequency distributions in single and multiple BCC; $\chi^2_3 = 10.49$, $P = 0.015$. **Frequency GSTM1 null + ♂ in single and multiple BCC; $\chi^2_1 = 9.44$, Yates corrected $P = 0.002$.

shown). Frequency distributions of GSTM1 A/B/CYP2D6 EM in controls and multiple BCC were significantly different (Table II). This difference largely resulted from the reduced frequency of subjects with CYP2D6 EM/GSTM1 A/B in the multiple BCC group compared with controls (Table II).

### *Interactions between gender and GSTM1, GSTT1 and CYP2D6 genotypes*

The proportion of males in the multiple BCC group (61.3%) was significantly greater than in controls (47.0%) ($\chi^2_1 = 11.85$, Yates corrected $P = 0.0006$) and single BCC (52.2%) ($\chi^2_1 = 5.29$, Yates corrected $P = 0.0214$, odds ratio 1.45, 95% CI 1.05–2.00). Interactions between GSTM1 null and male gender were examined by comparing multinomial frequency distributions (Table III); distributions in single and multiple BCC were significantly different and, the frequency of the combination GSTM1 null/male gender was significantly greater in patients with multiple tumours (odds ratio 1.72, 95% CI 1.21–2.44).

### *Interactions between patient characteristics and genotypes*

The proportions of patients in the single and multiple BCC groups with brown, blue or green eyes were not significantly different (data not shown).

Frequency distributions of skin types 1–5 in the single and multiple BCC cases were also not significantly different (Table IV-VI). Considering skin type in terms of no protection (type 1) and variable protection to UV (types 2–5), we compared multinomial frequency distributions of GSTM1 null with skin type 1 in the patients with single and multiple BCC. The proportion of subjects with these factors was significantly greater in the multiple BCC group than in those with a single BCC (Table IV-VI). Thus, the frequency distributions of GSTM1 null/skin type 1 were significantly different and, the proportion of subjects who were GSTM1 null with skin type 1 was significantly greater (Table IV-VI; odds ratio 3.25, 95% CI 1.30–8.27) in the multiple BCC group. We examined the data for interactions between GSTM1 null/skin type 1/male gender by comparing multinomial frequency distributions of these factors in the single and multiple BCC groups. The distributions were almost significantly different (Table IV-VI). No significant interactions between GSTT1 null or CYP2D6 EM and skin type 1 were identified (data not shown).

### *Interactions between smoking and genotypes*

The proportion of cases who were current smokers or ever smokers was not significantly different in the single and multiple BCC groups. Comparisons of multinomial frequency

Table IV Skin type frequencies in single and multiple BCC

| Skin type | 1 | 2 | 3 | 4 | 5 |
|---|---|---|---|---|---|
| Single BCC ($n$ = 302) | 34 (11.3%) | 94 (31.1%) | 98 (32.5%) | 59 (19.5%) | 17 (5.6%) |
| Multiple BCC ($n$ = 162) | 26 (16.0%) | 50 (30.9%) | 54 (33.3%) | 27 (16.7%) | 5 (3.1%) |

Table V Frequency distributions of GSTM1 null and skin type 1

| | Controls (%) | Single BCC[a] (%) | Multiple BCC (%) |
|---|---|---|---|
| GSTM1 null + type 1 | 2 (4.4) | 9 (3.3) | 15 (10.0)[b] |
| Type 1 only | 0 (0) | 20 (7.4) | 7 (4.7) |
| GSTM1 null only | 24 (53.0) | 131 (48.2) | 66 (44.0) |
| Neither | 19 (42.2) | 112 (41.2) | 62 (41.3) |
| Total | 45 (100) | 272 (100) | 150 (100) |

[a]Frequency distributions in single and multiple BCC; $\chi^2_3 = 9.06$; $P = 0.0285$.
[b] Frequency GSTM1 null + skin type 1 in single *v* multiple BCC; $\chi^2_1 = 6.87$; $P = 0.009$.

Table VI Interactions between GSTM1 null, skin type 1 and male gender

| | Single BCC[a] (%) | Multiple BCC (%) |
|---|---|---|
| GSTM1 null + type 1 + male | 5 (1.9) | 7 (4.8) |
| GSTM1 null + type 1 only | 5 (1.9) | 7 (4.8) |
| Type 1 + male only | 10 (3.7) | 3 (2.0) |
| GSTM1 null + male only | 60 (22.2) | 44 (29.9) |
| Male only | 63 (23.3) | 34 (23.1) |
| Skin type 1 only | 10 (3.7) | 4 (2.7) |
| GSTM1 null only | 69 (25.6) | 22 (15.0) |
| None | 48 (17.8) | 26 (17.7) |

[a] Frequency distributions in single and multiple BCC; $\chi^2_7 = 13.88$, exact $P = 0.051$.

distributions of smoking with each of the GSTM1 null, GSTT1 null and CYP2D6 EM genotypes identified no differences between patients with single and multiple tumours (data not shown).

### Discussion

The role of factors other than UV in the pathogenesis of BCC is evident from work using a variety of experimental approaches (Heagerty *et al.*, 1994; Karagas *et al.*, 1994; Wei *et al.*, 1994; McHenry *et al.*, 1995). We have described further studies on the influence of allelism at loci encoding phase I and II detoxifying enzymes on susceptibility to this tumour. Genotype frequencies in controls have been compared with those in the total BCC group and patients with single and multiple carcinomas. Interactions with other relevant factors such as skin type, gender and smoking have also been studied.

The present study confirms, in a substantially larger patient group, previous work from this laboratory showing the heterozygote GSTM1 A/B genotype is associated with a reduced risk of multiple BCC (Heagerty *et al.*, 1994). The mechanism for this protective effect against multiple BCC is unclear but is presumably related to the ability of these enzymes to catalyse the metabolism of a variety of products of oxidative stress formed after exposure to UV and/or constituents of cigarette smoke and other environmental pollutants (Ketterer *et al.*, 1993). The finding that protection is associated with GSTM1 A/B but not GSTM1 A or GSTM1 B (largely *GSTM1*0* heterozygotes) suggests a gene dosage effect that is specific to multiple BCC but not other skin malignancies such as squamous cell cancer or malignant melanoma. No protective effect for GSTT1 was identified, although the genotyping assay used cannot differentiate *GSTT1*A/GSTT1*A* homozygotes and *GSTT1*0/GSTT1*A* heterozygotes. It is possible the minority of subjects (about 30%) with two expressed alleles are protected but this effect is diluted by the larger number of *GSTT1*0* heterozygotes.

We also found no differences in frequency distributions of CYP2D6 genotypes in the cases and controls though the frequency of CYP2D6 PM was greater in patients with single BCC than in controls.

Recent studies showing the interactive effects of GSTM1 and CYP1A1 genotypes suggest that the influence of detoxifying enzymes in mediating cancer risk will depend on allelism at other relevant loci (Nakachi *et al.*, 1993; Warwick *et al.*, 1994). We identified no significant interactions between the putatively poor detoxification genotypes, GSTM1 null, GSTT1 null and CYP2D6 EM but did find significant differences between controls and patients with multiple BCC in the frequency of the combinations GSTM1 A/B with CYP2D6 EM and, GSTM1 A/B with GSTT1 expressers.

The importance of GSTM1 was emphasised by the finding that the frequency of the combination GSTM1 null/skin type I was significantly increased in patients with multiple BCC compared with those with a single tumour. Skin type is an arbitrary and subjective classification of individual response to UV. The classification of skin type 1 defines an extreme sensitivity to UV, which results in an inflammatory response but no pigmentary response (Fitzpatrick *et al.*, 1988). Our results show GSTM1 null alone is not a significant determinant of development of multiple BCC but the influence of skin type 1 is synergistic, such that in combination they are a significant predisposing factor to multiple BCC, possibly because these individuals are relatively less able to cope with the chemical products of UV or those of the resulting inflammation.

Significant interactions between GSTM1 null and male gender were also identified. The incidence of non-melanoma skin cancer is higher in men than women and Karagas *et al.* (1994) showed that in males with a prior tumour, the risk of a further BCC is 50% greater than in women. We also found a greater proportion of men in the multiple tumour group than in the single BCC or control groups. The mechanism for the observed interactions between GSTM1 null and gender and skin type 1 is unclear. Females may be relatively protected because oestrogens appear to stimulate melanin production both *in vivo* and *in vitro* (McLeod *et al.*, 1994).

Previous studies have failed to demonstrate an association between smoking and BCC or, smoking and risk of further tumours (Hunter *et al.*, 1990; Karagas *et al.*, 1994). As the number of controls from whom a reliable smoking history could be obtained was limited, we did not compare the proportions of ever/never smokers in the case groups with those in controls. However, it is noteworthy that the proportion of smokers in our BCC case group was significantly greater ($P<0.0002$) than that found by the Health Promotion Service of the North Staffordshire Hospital during a survey of 1957 unmatched, local adults (465, 23.9%) questioned during 1993. In agreement with previous findings our data showed that smoking alone did not increase the risk of multiple tumours (Karagas *et al.*, 1994). We have now shown that smoking does not influence risk of multiple tumours even in combination with putatively poor detoxification genotypes.

A better understanding of factors that predispose to single and multiple BCC will help devise preventative strategies for what is an increasing public health problem. While we identified few factors that influence the development of a single BCC, factors that mediate susceptibility to multiple tumours were found. The importance of GSTM1 has been emphasised, both the protective effect of GSTM1 A/B and the increased risk associated with the combination of skin type 1 and male gender with GSTM1 null. The influence of GSTT1 and CYP2D6 appeared to be less significant except in combination with GSTM1 A/B. We believe that our results are compatible with the view that development of multiple tumours is not merely determined by time but rather, certain patients have a genetically mediated increased susceptibility (Karagas *et al.*, 1994). We also presume that our data have underestimated the differences between patients with single and multiple tumours as some patients with single BCC are likely to eventually develop further tumours. There are no data from British patients, although local clinical experience suggests that the frequency of multiple tumours is lower than that found in American studies. The significant interaction between GSTM1 and skin type 1 indicates that other polymorphic genes that influence this phenotype, such as those determining melanin production and the immune response, are promising candidates.

**Acknowledgements**
We gratefully acknowledge the support of the Cancer Research Campaign (project grant SP2207/0201).

**References**

FARNDON PA, DEL MASTRO RG, EVANS DG AND KILPATRICK MW. (1992). Location of gene for Gorlin syndrome. *Lancet*, **339**, 581–582.

FITZPATRICK TB. (1988). The validity and practicality of sun reaction skin types 1 through VI. *Arch. Dermat.* **124**, 869–871.

FRYER AA, ZHAO L, ALLDERSEA J, PEARSON WR AND STRANGE RC. (1993). Use of site-directed mutagenesis of allele-specific PCR primers to identify the GSTM1 A, GSTM1 B, GSTM1 A,B and GSTM1 null polymorphisms at the glutathione S-transferase, GSTM1 locus. *Biochem. J.*, **295**, 313–315.

GOUGH AC, MILES JS, SPURR NK, MOSS JE, GAEDIGK A EICHELBAUM M AND WOLF CR. (1990). Identification of the primary gene defect at the cytochrome $P_{450}$ CYP2D6 locus. *Nature*, **347**, 773–776.

HEAGERTY AHM, FITZGERALD D, SMITH A, BOWERS B, JONES P, FRYER AA, ZHAO L, ALLDERSEA J AND STRANGE RC. (1994). Glutathione S-transferase GSTM1 phenotypes and protection against cutaneous malignancy. *Lancet*, **343**, 266–268.

HUNTER DJ, COLDITZ GA, STAMPFER MJ, ROSNER B, WILLETT WC AND SPEIZER FE. (1990). Risk factors for basal cell carcinoma in a prospective cohort of women. *Ann. Epidemiol.*, **1**, 13–23.

JUGERT FK, AGARWAL R, KUHN A, BICKERS DR, MERK HF AND MUKHTAR H. (1994). Multiple cytochrome P450 isoenzymes in murine skin: Induction of P4501A, 2B, 2E and 3A by dexamethasone. *J. Invest. Dermat.*, **102**, 970–975.

KARAGAS MR. for the Skin Cancer Prevention Study Group. (1994). Occurrence of cutaneous basal cell and squamous cell malignancies among those with a prior history of skin cancer. *J. Invest. Dermat.*, **102**, 10S–13S.

KARAGAS MR AND GREENBERG ER. (1995). Unresolved issues in the epidemiology of basal cell and squamous cell skin cancer. In *Skin Cancer: Mechanisms and Human Relevance*, Mukhtar H (ed.) pp. 79–86. CRC Press: Boca Raton, FL.

KETTERER B, TAYLOR J, MEYER D, PEMBLES S, COLES B, CHULIN X AND SPENCER S. (1993). Some functions of glutathione transferases. In *Structure and Function of Glutathione Transferases*. Tew K, Mannervik B, Mantle TJ, Pickett CB and Hayes JD (eds) pp. 15–27. CRC Press: Boca Raton, FL.

KRICKER A, ARMSTRONG BK, JONES ME AND BURTON RC. (1993). *Health, Solar UV Radiation and Environmental Change*. Technical Report no. 13, pp. 52–61. IARC: Lyon.

MCHENRY PM, AITCHISON T AND MACKIE RM. (1995). Comparison of risk factors for lentigo maligna melanoma, basal cell carcinoma and squamous cell carcinoma. *Scot. J. Med.*, (in press).

MCLEOD SD, RANSON M AND MASON RS. (1994). Effects of estrogens on human melanocytes *in vivo*. *J. Steroid Biochem. Mol. Biol.*, **49**, 9–14.

NAKACHI K, IMAI K, HAYASHI S AND KAWAJIRI K. (1993). Polymorphisms of the CYP1A1 and glutathione S-transferase genes associated with susceptibility to lung cancer in relation to cigarette dose in a Japanese population. *Cancer Res.*, **53**, 2994–2999.

PEMBLE S, SCHROEDER KR, SPENCER SR, MEYER DJ, HALLIER E, BOLT HM, KETTERER B AND TAYLOR JB. (1994). Human glutathione S-transferase theta (GSTT1): cDNA cloning and the characterisation of a genetic polymorphism. *Biochem J.*, **300**, 271–276.

SEIDEGARD J, VORACHEK WR, PERO RW AND PEARSON WR. (1988). Hereditary differences in the expression of the human glutathione S-transferase activity on trans-stilbene oxide are due to a gene deletion. *Proc. Natl Acad. Sci. USA*, **85**, 7293–7297.

STRANGE RC. (1993). The glutathione S-transferase GSTM1 locus and cancer susceptibility. In *Structure and Function of Glutathione Transferases*, Tew K, Mannervik B, Mantle TJ, Pickett CB and Hayes JD. (eds) pp. 160–171. CRC Press: Boca Raton, FL.

WARWICK AP, SARHANIS P, REDMAN C, PEMBLE S, TAYLOR J, KETTERER B, JONES P, ALLDERSEA J, GILFORD J, YENGI L, FRYER AA AND STRANGE RC. (1994). Theta class glutathione S-transferase GSTT1 genotypes and susceptibility to cervical neoplasia: Interactions with GSTM1, CYP2D6 and smoking. *Carcinogenesis*, **15**, 2841–2845.

WEI Q, MUTANOSKI GM, FARMER ER, HEDAYATI MA AND GROSSMAN L. (1994). DNA repair related to multiple skin cancers and drug use. *Cancer Res*, **54**, 437–440.

WEINSTOCK MA. (1994). Epidemiologic investigation of nonmelanoma skin cancer mortality: The Rhode Island follow-back study. *J. Invest. Dermat.*, **102**, 6S–9S.

WOLF CR, SMITH CAD, GOUGH AC, MOSS JE, VALLIS KA, HOWARD G, CAREY FJ, MILLS K, MCNEE W, CARMICHAEL J AND SPURR NK. (1992). Relationship between the debrisoquine polymorphism and cancer susceptibility. *Carcinogenesis*, **13**, 1035–1038.